# Depression, anxiety and posttraumatic stress disorder six months following preeclampsia and normotensive pregnancy: a P4 study

**DOI:** 10.1186/s12884-022-04439-y

**Published:** 2022-02-07

**Authors:** Lynne Roberts, Amanda Henry, Samuel B. Harvey, Caroline S. E. Homer, Gregory K. Davis

**Affiliations:** 1 Women’s and Children’s Health, St George Hospital, Sydney, Australia; 2 St George and Sutherland Clinical School, University of NSW, Sydney, Australia; 3 School of Women’s and Children’s Health, UNSW Medicine, University of NSW, Sydney, Australia; 4 The George Institute, Sydney, Australia; 5 The Black Dog Research Institute, Sydney, Australia; 6 Faculty of Medicine, University of NSW, Sydney, Australia; 7 Burnet Institute, Maternal, Child and Adolescent Health Program, Melbourne, Australia; 8 Faculty of Health, University of Technology Sydney, Sydney, Australia

**Keywords:** Preeclampsia, Depression, Anxiety, Posttraumatic stress disorder, Mental health, Postpartum

## Abstract

**Background:**

Mental health is an integral part of overall health. Mental health disorders following childbirth are common and poor maternal mental health has consequences for both the mother and her infant. Preeclampsia is also relatively common in pregnancy but there is little known about the intersection between these two important conditions. Gaining a better understanding of the psychological consequences following preeclampsia is important, especially the link with depression, anxiety and posttraumatic stress disorder. If women who experience preeclampsia are recognised as being at increased risk of poor mental health, targeted screening in the postpartum period should be implemented.

**Aims:**

To describe the prevalence and symptom severity of depression, anxiety and posttraumatic stress disorder at six months postpartum in women, who had a diagnosis of preeclampsia, compared to those who had normal blood pressure in pregnancy.

**Methods:**

The mental health component of the prospective cohort study, the Postpartum, Physiology, Psychology and Paediatric follow-up study (P4 Study) was used. Women diagnosed with preeclampsia (*n* = 90) and those who were normotensive during pregnancy (*n* = 302) completed the Edinburgh Postnatal Depression Scale, General Anxiety Disorder Scale, and the Posttraumatic Stress Diagnostic Scale or Posttraumatic Stress Diagnostic Sclae-5 at six months postpartum.

**Results:**

At six months postpartum, depressive scores were similar in both groups but a higher proportion of women from the preeclampsia group scored above the threshold for depression (2% v 7% *p* = 0.04). There were no differences between the groups in the prevalence or severity of anxiety or PTSD. However, more women in the preeclampsia group reported their birth experience as a traumatic event (1% vs 7%, *p* = 0.01). On correlation testing and modelling, booking Edinburgh Postnatal Depression Scale score, any mental health history, experiencing birth as traumatic and the General Anxiety Disorder Scale score were independent predictors of postpartum Edinburgh Postnatal Depression Scale scores.

**Conclusion:**

The postpartum clinical care of women with preeclampsia often focusses on the immediate physical health issues, but these women may also benefit from mental health screening. Targeted screening of preeclamptic women in the postpartum period may lead to more timely referral and initiation of treatment.

**Trial registration:**

Retrospectively registered on 18/11/2013 with the Australian and New Zealand Clinical Trials Registry. Registration Number: ACTRN12613001260718.

## Background

Mental health is an integral part of overall health and is defined by the World Health Organization as a state of well-being where individuals realise their abilities, cope with the normal stress of life, work effectively, and are able to contribute to their community [1]. Poor mental health can impact on all aspects of life, including work performance, carrying out everyday activities, relationships with family and friends and being a mother [1].

Pregnancy and childbirth is stressful and worrisome for many women [2] and mental health disorders following childbirth are common. Worldwide, one in seven women experience depression in the year following birth and one in five experience anxiety, commonly in combination with depression, during the same period [3]. The global prevalence of postpartum posttraumatic stress disorder (PTSD) is less certain and has been reported as 1–2% [4]. However, a literature review on birth experience [5] reported that 20–48% of women describe their birth as a traumatic event, meaning the true incidence of posttraumatic mental health disorder, including PTSD, could be much higher. For many women, pregnancy and childbirth is a complex experience and may lead to a life threatening situation for themselves and/or their baby [6, 7] which may elicit a variety of psychological responses [8]. An example of such a pregnancy is one complicated by a hypertensive disorder.

Hypertensive disorders of pregnancy (HDP) occur in 5–10% of pregnancies [9] and are one of the leading causes of maternal and perinatal morbidity and mortality [10]. One of the HDP is preeclampsia, a multi-system disorder with new-onset hypertension after 20 weeks gestation and evidence of involvement of at least one maternal organ system (renal, hepatic, haematological, neurological) and/or the unborn baby due to uteroplacental dysfunction (e.g. fetal growth restriction) [9, 11]. There are well documented long term physical health consequences for women following HDP such as cardiovascular disease (hypertension, stroke, ischaemic heart disease), kidney disease, and diabetes [12–16], however little is known about women's mental health following this complication.

Previous studies into maternal mental health following HDP are inconclusive as they have shown mixed and conflicting results, possibly due to their heterogeneity. One systematic review [17] suggested that despite results being mixed, there appeared to be an association between preeclampsia and depression, no association with anxiety, and possibly a link to PTSD. Another systematic review specifically investigating PTSD following pregnancy complications, [18] including but not limited to preeclampsia, suggested that there may be a link particularly when there are poor neonatal outcomes. A more recent literature review on the relationship between HDP and depression, anxiety and PTSD [19] concluded that results were conflicting and inconsistent, and although some studies reported no significant association, there was a trend towards increased prevalence and symptom severity of depression, anxiety and PTSD following HDP, particularly following the more severe presentations of preeclampsia.

Gaining a better understanding of the psychological consequences of pregnancies complicated by preeclampsia is important for several reasons. Firstly, poor mental health can negatively impact maternal and infant health. In addition to the effect on a woman’s emotional welfare and daily functioning, poor mental health may impair her nurturing ability and the formation of a relationship with her baby [20]. Secondly, long term and/or untreated poor maternal mental health have been associated with poor infant wellbeing, particularly with regard to behaviour and cognitive development [21]. In severe cases, women with postpartum depression may commit suicide [22] and in those women with psychotic illnesses, the risk of infanticide, though rare, must not be overlooked [22].

In the postpartum period, poor mental health is often undetected leading to a delay in, or a lack of, treatment [23]. Guidelines emphasise the importance of implementing interventions targeting women displaying the early signs and symptoms of poor mental health [24–26], however, many women who would benefit from intervention are not identified in the postpartum period. Early intervention is critical for preventing or reducing the progress of poor mental health in the perinatal period [23]. If women who experience preeclampsia are recognised as being at increased risk of poor mental health, targeted screening early in the postpartum period could lead to more timely referral and treatment initiation. The aim of this study was therefore to investigate the prevalence and symptom severity of depression, anxiety and PTSD at six months postpartum, in women who had a diagnosis of preeclampsia, compared to women who had normal blood pressure in pregnancy.

## Methods

A component of the prospective cohort study, the Postpartum, Physiology, Psychology and Paediatric follow-up study (P4 Study) being undertaken at St George Hospital, a metropolitan teaching hospital located in southeast Sydney, Australia. The full methodology of the P4 study has been described previously [27]. The P4 Study was approved by the South East Sydney Local Health District (SESLHD) Human Research Ethics Committee (HREC ref no: 12/195) and Governance approval was obtained from SESLHD (SSA ref: 12/G/224). All study methods were carried out in accordance with the approved study protocol and regulations. Informed written consent was obtained from each woman at the six month study visit, prior to any study procedure taking place.

The study population consisted of women who gave birth between January 2013 and December 2018 and either had normal blood pressure during pregnancy, or were diagnosed with preeclampsia by specialist medical staff as per International Society for the Study of Hypertension in Pregnancy (ISSHP) guidelines, which incorporate non-proteinuric preeclampsia and include fetal growth restriction as potential diagnostic criteria [9]. At six months postpartum, demographic data were collected from the medical record along with pregnancy, labour and birth details. Mental health data were collected directly from the women at their six month P4 Study visit. Women completed a structured questionnaire that included three self-reporting instruments; the Edinburgh Postnatal Depression Scale (EPDS) [28], the General Anxiety Disorder (GAD-7) Scale [29], and the Posttraumatic Stress Disorder Scale (PDS) [30] or the PDS-5 [31] to screen for depression, anxiety and PTSD. Each instrument was selected for reliability, validity and ease of use in the research setting.

### Edinburgh Postnatal Depression Scale (EPDS)

The EPDS is a 10-item instrument [32]. Women were asked to select the most appropriate of four responses for each statement that best described how they had been feeling during the past seven days. Responses are scored from 0–3 with a possible total score of 0–30, higher scores signifying more depressive symptoms. A cut off score of greater than 12 was used as the threshold for significant symptoms. A moderate score of 10–12 was also compared between groups.

### General Anxiety Disorder 7 (GAD-7) Scale

The GAD-7 is used for screening and assessing the severity of generalised anxiety disorder (GAD) [33–35]. It comprises seven items that describe prominent features of generalised anxiety, such as, excessive worry and irritability. Responses are scored from 0–3 with a possible total score of 0–21. Cut off scores of 5, 10, and 15 represent mild, moderate and severe anxiety levels, respectively [35, 36]. In this study, a score of 10 or greater was used as the threshold for significant symptoms.

### Posttraumatic stress Diagnostic Scale (PDS) and PDS-5

Two instruments were used to screen for PTSD. The initial instrument, the PDS [37], was superseded in 2015 by the PDS-5 [38] to align with changes made to the diagnostic criteria for PTSD [39]. Both instruments include a checklist of traumatic events followed by a scoring system on the PTSD symptoms the respondent had experienced. These instruments are not specifically designed to be used following childbirth, however, they can be used following exposure to any traumatic event by using the “other” category in the list of stressors [40].

The two instruments varied in symptom clusters, scoring methods and possible total scores, making it difficult to unify the results. Therefore, total scores were converted to a percentage of the possible total score for each instrument (51 for the PDS and 80 for the PDS-5). The prevalence of significant PTSD symptoms was calculated from each PDS version; meeting every criteria A-F for the PDS and a score greater than 28 for the PDS-5.

If the score on any of the screening instruments met the cut off threshold, the woman was counselled, and if necessary a referral was made to either her general practitioner or the perinatal mental health service at the hospital for follow up. Referral was undertaken if she did not have existing mental health support, according to her preference, and with her consent.

### Statistical analysis

Data analysis was undertaken using SPSS V26. Data were initially analysed descriptively. Data are presented as number (percentage) for proportions, as mean (standard deviation) for parametric data, and median (interquartile range) for non-parametric data. Comparisons were made between the normotensive and preeclampsia groups, with Chi-squared or Fisher’s exact test for comparison of categorical variables and independent samples t-test or Mann–Whitney-U for comparison of parametric and non-parametric continuous and ordinal data. Spearman’s correlation testing was used to explore associations between the major mental health outcomes (EPDS six months postpartum and GAD-7 score six months postpartum) with demographic characteristics, past history including mental health, and pregnancy and neonatal outcomes. Logistic regression modelling was then performed, (outcome variables were the six months postpartum EPDS and six months postpartum GAD-7) incorporating the major demographic factors known to influence mental health (maternal age, parity, BMI, relationship status), pregnancy outcome and early postpartum factors which may influence mental health including preeclampsia, gestation at birth, maternal acute care admission, baby SCN/NICU admission, caesarean birth, and breastfeeding status at six months postpartum, and other markers of past or current mental health (traumatic birth, history of any mental health disorder, booking EPDS). Predictors were examined individually, in groups, and with forced entry to final model and use of bootstrapping.

A-priori power calculation was not performed, as the overall P4 study was powered on (a) producing a reference range for maternal blood pressure six months postpartum amongst normotensive pregnancy women (b) detecting differences in the proportion of preeclamptic women outside reference range for blood pressure six months postpartum [41]. For comparative tests, statistical significance was set at *p* value < 0.05. As this was an exploratory study, adjustment for multiple comparisons was not made.

## Results

A total of 392 women participated, 302 in the normotensive group and 90 in the preeclampsia group. Women attended their six month P4 Study visit at an average of 27 weeks postpartum (SD, 1.3).

Baseline characteristics of the participating women are summarised in Table 1. Women in both groups were from similar ethnic backgrounds and the majority were in a relationship. The women in the preeclampsia group were slightly younger (*p* = 0.02) and significantly more joined the study after having their first baby (*p* < 0.001), compared to the normotensive group.

**Table 1:** Demographic details by group

	Normotensive Group *N* = 302			Preeclampsia Group *N* = 90			p-value
	N	%	Mean (SD) or median (IQR)	N	%	Mean (SD) or median (IQR)	
Age (years)			33 (5)			32 (5)	**0.02**
First baby	151	50		66	73		** < 0.001**
Ethnicity							
-White	162	54		47	52		0.15
-Asian	67	22		18	20		
-ATSI	2	0.7		2	2		
-Polynesian	1	0.3		3	3		
-European	41	14		11	12		
-Other	28	9		9	10		
Highest education							
-University degree	204	68		54	60		**0.04**
-Trade-Certificate	70	23		32	36		
-Secondary school	26	9		4	4		
In a relationship	296	98		88	98		0.89
Booking EPDS score (*n* = 282/80)			3 (1–5)			5 (2–7)	**0.01**
Booking EPDS > 12	7	3		5	6		0.15
History of mental health disorder	65	22		20	22		0.89

*ATSI* Aboriginal or Torres Strait Islander, *EPDS* Edinburgh Postnatal Depression Scale

In keeping with their complicated pregnancies, women in the preeclampsia group experienced more intervention during labour and birth compared to women in the normotensive group (Table 2). The mode of birth differed significantly; more normal vaginal births in the normotensive group, more caesarean births in the preeclampsia group. Following the birth, four women after normotensive pregnancy (1%) required admission to an acute care unit, all for postpartum haemorrhage management, compared with 25 (28%) from the preeclampsia group (monitoring of preeclampsia including magnesium sulphate infusion, blood pressure control, and postpartum haemorrhage). There were more preterm births (< 37 weeks) in the preeclampsia group, with a significantly larger proportion of babies requiring admission to either the Special Care Nursery (SCN) or Neonatal Intensive Care Unit (NICU).

**Table 2:** Labour and birth outcomes by group

	Normotensive Group *N* = 302			Preeclampsia Group *N* = 90			p-value
	N	%	Mean (SD)	N	%	Mean (SD)	
Gestation at birth (weeks)			39 (2)			37 (3)	** < 0.001**
< 32 weeks	2	1		4	4		**0.03**
32–37 weeks	17	6		26	29		** < 0.001**
Labour							** < 0.001**
- Spontaneous	175	58		8	9		
- Induction	91	32		59	65		
- No labour	30	10		23	26		
Mode of birth							** < 0.001**
- Normal Vaginal	198	66		29	32		
- Assisted Vaginal	46	15		17	19		
- Elective Caesarean Section	24	8		9	10		
- Emergency Caesarean section	34	11		35	39		
Maternal postpartum acute care admission	4	1		25	28		** < 0.001**
SCN admission	39	13		48	53		** < 0.001**
NICU admission	6	2		7	8		**0.007**
Feeding at maternal postpartum discharge							**0.006**
-Breast feeding/expressing	283	94		76	84		
-Mixed AF/BF	10	3		10	11		
-Formula	9	3		3	3		
Breast feeding at 6 months							** < 0.001**
- No, didn’t breast feed	13	4		3	3		
- No, stopped now	43	14		32	36		
- Yes, still breast feeding	246	82		55	61		

*AF* artificial feed, *BF* breast feed, *NICU* Neonatal Intensive Care Unit, *SCN* Special Care Nursery

At six months postpartum, the full cohort (*N* = 392) completed the EPDS and GAD-7, 383 completed the PDS or PDS-5 (293 normotensive, 90 preeclampsia) (Table 3). The PDS was out of print/unavailable at study commencement, hence the first nine recruited women (all normotensive) did not complete it. The prevalence and symptom severity for depression and anxiety were low and similar between the groups. The only statistically significant difference was a higher proportion of women after preeclampsia scoring greater than 12 on the EPDS (*p* = 0.04). For PTSD, although there was no difference in prevalence or symptom severity, a greater proportion of women in the preeclampsia group reported their birth as being a traumatic event (*p* = 0.01). There was no difference between groups in the number of women who sought help from their GP or who were referred for specialist mental health care at any time in the first six months postpartum prior to their P4 Study visit.

**Table 3:** Mental health at 6 months postpartum by group

	Normotensive Group *N* = 302			Preeclampsia Group *N* = 90			
	N	%	Mean (SD) or median (IQR)	n	%	Mean (SD) or median (IQR)	*p*-value
Depression							
-EPDS score			3 (2–6)			4 (2–7)	0.10
-EPDS > 12	7	2		6	7		**0.04**
-EPDS 10–12	19	6		4	4		0.51
-EPDS 10 or higher	26	9		10	11		0.47
Anxiety							
-GAD-7 score			2 (0–3)			2 (0–3)	0.25
-GAD-7 ≥ 10	9	3		6	7		0.12
PTSD (n = 293/90)							
-Score as a %			7 (11)			8 (15)	0.59
-Met criteria	5	2		2	2		0.75
-Birth reported as traumatic event	4	1		6	7		**0.01**
Seen GP re mental health prior to P4 visit	15	5		7	8		0.31
Specialist/psychologist referral prior to P4 visit	17	6		6	7		0.71

*EPDS* Edinburgh Postnatal Depression Scale, *GAD-7* General Anxiety Disorder Scale, *GP* General Practitioner, *PTSD* Posttraumatic Stress Disorder

The six month EPDS was most strongly correlated with six month GAD-7 score (r = 0.65, *p* < 0.001). It was also significantly correlated with booking EPDS (r = 0.46, *p* < 0.001), having any prior mental health history, (r = 0.27, *p* < 0.001) and meeting criteria for PTSD (r = 0.16, *p* = 0.002). There were no significant correlations with any demographic or pregnancy and neonatal outcome factors, including preeclampsia status (r = 0.08, *p* = 0.1). For GAD-7, as well as strong, significant correlation with six month EPDS, GAD-7 score six months postpartum was correlated with booking EPDS (r = 0.34, *p* < 0.001), having any mental health history (r = 0.35, *p* < 0.001), and meeting criteria for PTSD (r = 0.17, *p* = 0.001). The GAD-7 score was also not significantly correlated with demographic factors, pregnancy/neonatal outcome, or preeclampsia status.

The multivariate analysis confirmed the importance of the mental health factors noted on correlation testing, and minimal contribution of demographic, pregnancy/neonatal outcome, and maternal preeclampsia status to explaining variability in EPDS and GAD-7 scores. For the six month postpartum EPDS, booking EPDS, any maternal mental health history, GAD-7 at six months, and experience of traumatic birth explained 56.2% of variability (R2 = 0.562, *p* < 0.001), with marginal improvement on addition of all variables including preeclampsia status to the final model, which explained 57.9% of variability. For GAD-7, the mental health variables alone explained 55% of variability, and the final model 56.9% of variability (R2 = 0.569, *p* < 0.001).

## Discussion

This study focused on the mental health disorders depression, anxiety and PTSD in women who experienced preeclampsia and those who were normotensive in pregnancy. As expected, women in the preeclampsia group had more intervention during the labour and birth, gave birth at an earlier gestation, and required more postpartum acute care admissions and their babies required more nursery admissions. These are all characteristic of pregnancies complicated by preeclampsia [42]. Unsurprisingly, women who experienced preeclampsia were more likely to describe their birthing experience as traumatic. Importantly, this study has been able to demonstrate that this did not translate into increased rates of PTSD six months after the birth. Experiencing preeclampsia during pregnancy did increase women’s rate of postpartum depression (*p* = 0.043) however after adjustment in a multivariate analysis, there were no significant correlations with any demographic or pregnancy and neonatal outcome factors, including preeclampsia status, but was correlated with the pregnancy booking EPDS and having a prior mental health disorder. These results have important implications for maternity care providers managing preeclampsia in pregnancy and for interventions aimed at identifying women at high risk of postpartum mental health problems.

Previous studies have reported the prevalence of depression in women following HDP to range from 7 to 39% [43–47]. In addition, some studies have shown that the prevalence of depression increased in line with an increase in the severity of preeclampsia symptoms [43, 45], others suggesting that preterm birth, where the baby requires nursery admission, increases the prevalence of depression, not the experience of preeclampsia [48]. In our study, the prevalence of depression following preeclampsia was reported at 7% which is on the low level of that previously reported, but significantly higher than that reported by the normotensive group (2%). This may be explained by differences in the study population characteristics, particularly the severity of the preeclampsia symptoms and gestation at birth. The majority of women in this study experienced later onset preeclampsia as it is protocol at the study site to transfer women to a tertiary centre if birth is expected prior to 32 weeks gestation.

Women who were high risk of developing postpartum depression were recognised as those who had preeclampsia, a higher score on the early pregnancy booking EPDS and a history of any mental health disorder. This is in line with several researchers who have reported that having a mental health disorder before pregnancy is an important risk factor for perinatal depression [49]. In contrast to other studies [17, 19], we found minimal contribution of demographic, pregnancy and neonatal outcomes to explaining the higher EPDS in the preeclampsia group.

As with depression, this study found a low prevalence of anxiety and PTSD in both the overall cohort and the preeclampsia group, compared to that reported in the literature. The prevalence of general anxiety disorder has been reported at 10% [50] to 13% [51] in the six month postpartum population and 26–31% following preeclampsia [44, 52]. A systematic review and meta-analysis [53], reported a PTSD prevalence of 4.9% at six months postpartum in the general population, and 16.6% at six months postpartum in women from a high-risk pregnancy group which included, but was not limited to, preeclampsia. The low prevalence of anxiety and PTSD in our study may be due to the characteristics of the study cohort or they could be attributed to the timing of the assessment.

Having a preterm birth has been associated with increased anxiety [54, 55] so it could be argued that the prevalence of anxiety would have been higher in this study if the very preterm gestations had been included. A qualitative study regarding birth experience following preeclampsia [55] suggested that most women voice worry and anxiety following a preeclampsia diagnosis. However, by the six month postpartum stage this worry dissipates as most women make significant adjustments to parenthood and feel reassured by their health and their baby’s health, over time. This concept of transient anxiety following preeclampsia has been reported in another study [56] where significant improvement in screening instrument scores was found over time for all women except for those whose infant had major morbidity.

One of the difficulties of measuring PTSD is that the perception of a traumatic birth varies between women, is subjective and therefore can be difficult to define [5]. Some research reports a difference in PTSD prevalence between women who gave birth preterm and term, with or without preeclampsia, [48, 54], suggesting that the consequences of a preterm birth led to the symptoms of PTSD, rather than the preeclampsia. Other researchers have suggested that a traumatic birth experience stems from the woman’s observation of her treatment, complications including risks to her baby, and her perception of her choice and control over medical procedures [57, 58]. In our study there was no difference detected between the groups regarding women who scored above the threshold for probable PTSD. This is in contrast to studies included in a systematic review and meta-analysis [53] that reported higher rates of PTSD in women following HDP compared to women who were normotensive in pregnancy. It is possible that the women in the preeclampsia group had some protection from PTSD because of the collaborative continuity of care they received, but stressful situations which are not modifiable due to the nature of PE, may have contributed to some PTSD symptoms. This may explain why there were more reports of a traumatic birth from women in the preeclampsia group and why several women from this group reported symptoms of PTSD but did not meet the threshold for diagnosis.

Overall, this study did not show a statistically significant difference between the preeclampsia and normotensive groups in all areas of mental health studied except for a small difference in the proportion of women who scored above the threshold on the EDPS. However, the women in the preeclampsia group had a higher mean score on all the screening instruments, more met the threshold for anxiety and more reported their birth as a traumatic event. These results, although not statistically significant, are clinically important and there is a case for routine mental health screening in the postpartum period following preeclampsia, especially for those women who score high on the booking EPDS and/or have a history of poor mental health. This would facilitate early recognition of poor mental health, and early initiation of support and referral.

Strengths of this study include the prospective design, the use of validated screening instruments (EPDS, GAD-7, PDS and PDS-5) that have been proven to be accurate in the research setting compared to clinical interview and assessment, and the avoidance of some recall bias by collecting accurate data directly from the woman’s medical record. The diagnosis of preeclampsia was made, and subsequent clinical care was based on ISSHP guidelines and protocols leading to a consistent and accurate diagnosis and clinical management. Limitations include the women who volunteered to participate in the P4 Study were more likely to be in good mental health, more motivated and health literate compared to the general population which may have affected the results, there is little data from women who experienced preeclampsia less than 32 weeks gestation and a lack of data from women who gave birth less than 30 weeks gestation. Including these women may have given different results. Using two different versions of the PDS scale may have affected the results with 42% of the cohort completing the PDS (38% of the preeclampsia group and 43% of the normotensive group), the remaining completing the PDS-5. The timing of the follow-up may have missed capturing some postpartum mental health consequences. We may have missed changes in early distress as these may have resolved by six months postpartum and delayed distressed would have been missed if it occurred after six months. Lastly, women diagnosed with PE at the study site are cared for by a multidisciplinary continuity of care model which may have offered some protection from mental health symptoms.

## Conclusion

Women who experienced preeclampsia in their pregnancy reported low, and not significantly different anxiety and PTSD prevalence and symptom severity at six months postpartum compared to women who were normotensive during pregnancy. Significantly more women in the preeclampsia group met the threshold score for depression compared to the normotensive group. Having a history of a prior mental health disorder and a higher booking EPDS score were predictors of postpartum depression. The care of women with preeclampsia often focusses on the immediate physical health issues, but they may also benefit from mental health screening. Targeted screening of these women early in the postpartum period may be useful, particularly for those with a mental health history and/or high booking EPDS score, and lead to timelier referral and treatment initiation.

## Data Availability

The datasets generated and/or analysed during the current study are not publicly available due to limitations of ethical approval involving the patient data and anonymity but are available from the corresponding author on reasonable request.

## Abbreviations

ATSI: Aboriginal or Torres Strait Islander
EPDS: Edinburgh Postnatal Depression Scale
GAD-7: General Anxiety Disorder 7 Scale
GP: General Practitioner
HDP: Hypertensive Disorders of Pregnancy
ISSHP: International Society for the Study of Hypertension in Pregnancy
P4: Postpartum Physiology, Psychology and Paediatric follow up Study
PDS: Posttraumatic stress Diagnostic Scale
PDS-5: Posttraumatic stress Diagnostic Scale – 5
PTSD: Posttraumatic Stress Disorder
